# Genetic divergence of influenza A NS1 gene in pandemic 2009 H1N1 isolates with respect to H1N1 and H3N2 isolates from previous seasonal epidemics

**DOI:** 10.1186/1743-422X-7-209

**Published:** 2010-09-01

**Authors:** Giulia Campanini, Antonio Piralla, Stefania Paolucci, Francesca Rovida, Elena Percivalle, Giovanni Maga, Fausto Baldanti

**Affiliations:** 1 Molecular Virology Unit, Virology and Microbiology Dept, Fondazione IRCCS Policlinico San Matteo, 27100 Pavia, Italy; 2 National Research Center IGBE CNR, 27100 Pavia, Italy

## Abstract

**Background:**

The Influenza A pandemic sustained by a new H1N1 variant (H1N1v) started in Mexico and the USA at the end of April 2009 spreading worldwide in a few weeks. In this study we investigate the variability of the NS1 gene of the pandemic H1N1v strain with respect to previous seasonal strains circulating in humans and the potential selection of virus variants through isolation in cell culture.

**Methods:**

During the period April 27^th ^2009-Jan 15^th ^2010, 1633 potential 2009 H1N1v cases have been screened at our center using the CDC detection and typing realtime RT-PCR assays. Virus isolation on MDCK cells was systematically performed in 1/10 positive cases. A subset of 51 H1N1v strains isolated in the period May-September 2009 was selected for NS1 gene sequencing. In addition, 15 H1N1 and 47 H3N2 virus isolates from three previous seasonal epidemics (2006-2009) were analyzed in parallel.

**Results:**

A low variability in the NS1 amino acid (aa) sequence among H1N1v isolates was shown (aa identity 99.5%). A slightly higher NS1 variability was observed among H1N1 and H3N2 strains from previous epidemics (aa identity 98.6% and 98.9%, respectively). The H1N1v strains were closely related (aa identity 92.1%) to swine reference strain (A/swine/Oklahoma/042169/2008). In contrast, substantial divergence (aa identity 83.4%) with respect to human reference strain A/Brevig Mission/1/1918 and previous epidemic strains H1N1 and H3N2 (aa identity 78.9% and 77.6%, respectively) was shown. Specific sequence signatures of uncertain significance in the new virus variant were a C-terminus deletion and a T215P substitution.

**Conclusions:**

The H1N1v NS1 gene was more conserved than that of previous epidemic strains. In addition, a closer genetic identity of H1N1v with the swine than the human reference strains was shown. Hot-spots were shown in the H1N1v NS1 aa sequence whose biologic relevance remains to be investigated.

## Background

The 8^th ^segment of the influenza A virus genome encodes for two non structural proteins (NS1 and NS2) which were involved in virus immune evasion mechanisms. In particular, NS1 impairs the innate host immune response mediated by interferons (IFN) α and γ [1] and hampers the adaptive immune response by inhibiting the expression of TNF-α and IL-6 [2,3]. In addition, NS1 contributes significantly to the efficiency of virus replication through temporal regulation of virus mRNAs synthesis, control of the splicing process of the same mRNAs and the correct maturation and morphogenesis of virus particles [4]. Finally, the carboxy-terminal PDZ-ligand (PL) domain endows NS1 with the ability to play an important role in the compartimentalization of viral protein in the nuclei of infected cells [5,6].

In the last century, three Influenza A pandemics sustained by virus variants with divergent hemagglutinin (HA) and neuraminidase (NA) genes have occurred, with a major morbidity and mortality burden. In particular, the 1918 pandemic (Spanish Influenza) was sustained by a H1N1 strain, the 1957 pandemic (Asian Influenza) was caused by a H2N2 strain and the 1968 pandemic (Hong Kong Influenza) was triggered by the appearance of a reassorted H3N2 strain [7]. By contrast, the first pandemic of the new millenium was sustained by an Influenza A variant (H1N1v) with a complex genetic origin. The H1N1 swine lineage was established following introduction of the 1918/H1N1 avian virus in pigs. The novel H1N1v virus is a descendant of this original lineage, formed as a multiple reassortant of swine influenza viruses circulating in pigs in North America and Eurasia. Phylogenetic analysis was consistent with the hypothesis that this new virus circulated in pigs for at least a decade, before surfacing in the human population for the first time in Mexico, in January 2009 [8]. Although the H1N1v pandemic strain did not show significant morbidity and mortality, it rapidly spread worldwide. Another Influenza A virus raising major concern is the highly pathogenic avian H5N1 strain. This virus, first isolated in the Guangdong province of China in 1996, caused a small outbreak in humans in Hong Kong in 1997 [9], and is responsible for an ongoing pandemic in the avian population and occasional infections in humans (about 300 cases worldwide). While the H5N1 virus is not capable of human-to-human transmission and is only directly acquired from infected birds, its mortality in humans is very high (>60%) [10].

A number of studies on the pathogenicity mechanisms of the highly aggressive human A/1918/H1N1 and avian A/1997/H5N1 strains, revealed the important role of NS1 in mediating viral pathogenicity. Reverse genetics experiments showed that the NS1 protein of both viruses might be responsible for a lower susceptibility to the antiviral activity of IFN- and TNF-mediated responses [11-13]. NS1 is an highly conserved multifunctional protein, and its potential role in influenza virus pathogenicity has recently become evident. NS1 sequences can be grouped in two major alleles (A and B) [14,15]. Phylogenetic analysis revealed that all human, swine and equine influenza A viruses and a large number of highly pathogenic avian isolates all share the same NS1 allele (allele A) with similarity levels between 93% and 100% while allele B is present in the avian population only, suggesting a role in mammalian adaptation [4,16].

The present work was aimed at verifying: i) the variability of the NS1 gene of the pandemic H1N1v strain with respect to previous seasonal strains circulating in humans and ii) the potential selection of virus variants through isolation in cell culture.

## Materials and methods

### Specimens

As part of the National Influenza Surveillance Network (Influnet) and one of the two Lombardy Region virology reference centers, the Molecular Virology Unit, Fondazione IRCCS Policlinico San Matteo, received nasal swabs from the entire Lombardia Region (total population, about 10 million) for diagnosis and surveillance of the 2009 influenza A pandemic. Following national guidelines, in the period April-August, patients traveling from countries with widespread H1N1v infection and influenza-like illness (ILI) were individually screened. Following the pandemic declaration by WHO on June 11^th^, 2009, only patients with ILI admitted to the Policlinico San Matteo and other Hospitals in Lombardy were screened for influenza infection using the pan-influenza-A and the H1N1v-specific realtime RT-PCR assays developed by the Centers for Disease Control (CDC) (1600 Clifton Rd. Atlanta, GA, 30333, USA). Briefly, virus RNA was extracted from 200 μl nasal swab transport medium (Copan Diagnostics, Murrieta, CA, USA) using the NucliSens^® ^easyMAG^® ^automatic extraction (bioMérieux, Lyon, France) and eluted in 50 μl H_2_O. Five μl of extracted RNA were submitted to realtime RT-PCR using the Ag-Path-ID one-step RT-PCR kit (Applied Biosystems, Foster City, CA, USA), pan-Influenza A primers and probe targeting the highly conserved M gene, as well as primers and probe targeting a sequence of influenza A HA gene specific for H1N1v strain and the 7300 Real-Time PCR System (Applied Biosystems).

In addition, one out of ten real-time RT-PCR positive nasal swab samples were systematically inoculated onto Madin Darby canine kidney (MDCK) cells.

Forty-seven influenza A H3N2 and 15 H1N1 isolates recovered during the three preceeding seasonal epidemics were analyzed in parallel. Seasonal influenza A strains were detected, typed and recovered from nasopharyngeal aspirates (NPA) and bronchoalveolar lavage (BAL) samples from patients referred to the Policlinico San Matteo Hospital during the 2006-2007, 2007-2008 and 2008-2009 winter-spring seasons as previously reported [17-19].

### NS1 amplification and sequencing

Influenza A NS1 was amplified with subtype-specific primers using 5 μl of virus RNA extracted from 200 μl nasal swab, NPA and BAL samples as well as cell culture supernatants. In detail, "in house" and sequencing techniques were developed to obtain 822 bp amplicons (nt-25 to nt 797) using a H1N1v-specific primer set (forward, 5'-GCA AAA GCA GGG TGA CAA AAA C-3'; reverse, 5'-CTT CAA GCA GTA GTT GTA AGG C-3'), while H1N1- and H3N2-specific primer sets consisted of a common forward primer (5'-AGC AAA AGC AGG GTG ACA AAG A-3') and subtype-specific reverse primers (H1N1, 5'-AAC GTT CTA ATC TCT TGT TCC ACT TCA A-3'; H3N2, 5'-GAG AAA GTT CTT ATC TCC TGT TCC ACT-3') generating 845 (nt-26 to nt 819) and 848 (nt-26 to nt 822) bp amplicons, respectively. RT-PCR reactions were carried out using the Ag-Path-ID one-step RT-PCR kit (Applied Biosystems) in a GeneAmp^® ^PCR System 9700 thermal cycler (Applied Biosystems) using the following thermal profiles: i) H1N1v, 1 cycle at 45°C for 15 min and 95°C for 10 min, followed by 50 cycles at 95°C for 60 sec, 60°C for 50 sec and 72°C for 90 sec, with a final elongation of 7 min at 72°C; ii) H1N1 or H3N2, 1 cycle at 45°C for 15 min and 95°C for 10 min, followed by 50 cycles at 95°C for 60 sec, 55°C for 50 sec and 72°C for 90 sec, with a final elongation of 7 min at 72°C.

Sequencing of NS1 amplicons was performed with internal primers (H1N1v-forward, 5'-AGC CCT TAG TAG TAT CAA GGT C-3'; H1N1v-reverse, 5'-GCC TAG GCA AAA GAT AAT AGG C-3'; H1N1-forward,5'-CTC TTT GTG TCA GAA TGG ACC-3'; H1N1-reverse, 5'-AAT GTC AGA TTC TCC AAC CGG-3'; H3N2-forward, 5'-TTC ATG CTA ATG CCC AAG CAG-3'; H3N2-reverse, 5'-ACT ATG GTC TCT AGT CGG TC-3'), utilizing the ABI PRISM™ Big Dye Terminator chemistry (Applied Biosystems) and the ABI PRISM™ 3100 automatic sequencer (Applied Biosystems).

### NS1 sequence analysis

NS1 sequences were analyzed using the Sequencher 4.7 software (Gene Codes Corp., Ann Arbor, MI, USA). Multiple sequence alignments were obtained using the ClustalW 1.6 program and phylogenetic analysis was performed using the MEGA 4.0 program [20]. A neighbor-joining tree was generated using a Maximum Composite Likelihood method for simultaneously estimating evolutionary distances between all sequence pairs. Bootstrap analysis was performed using 1000 repetitions. Nucleotide sequences were deposited in the GenBank database [GenBank: HM745138-HM745250].

Intra- and Inter-strain analysis were performed by comparing mean values of nucleotide and amino acid identity between groups of sequences using the Student's *t*-test, while the analysis of variance was performed using the Bonferroni post-test.

## Results

### Specimens

During the period April 27^th ^2009-Jan 15^th ^2010, 1633 nasal swab samples were received for diagnosis of influenza A infection and surveillance of the pandemic. Of the tested specimens, 514 (31.5%) were positive for H1N1v. A total of 82 nasal swab samples (about one out of every ten consecutive positive) was inoculated onto MDCK cells, obtaining 53 virus isolates. Of these, 51 were submitted to NS1 sequence analysis.

### NS1 sequence analysis

**Figure 1:**
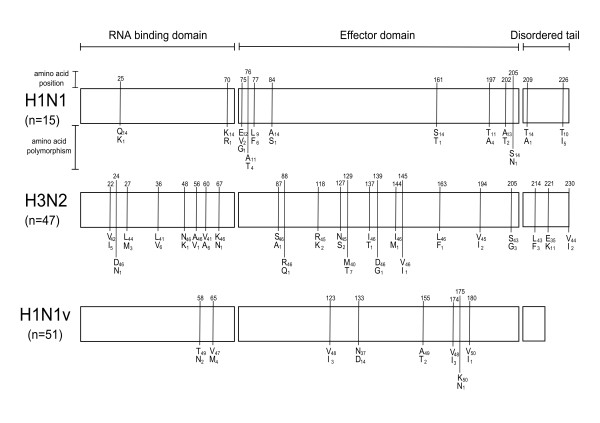
**Distribution of polymorphic codons in NS1 gene**. Schematic of influenza A NS1 gene domains and distribution of polymorphic codons in H1N1v isolates with respect to H1N1 and H3N2 isolates from previous seasonal epidemics.

With respect to 47 H3N2 and 15 H1N1 strains from previous epidemics, the H1N1v NS1 gene coding sequence is 33 nucleotides shorter, corresponding to a deletion of 11 amino acids at the C-terminus (690 nt and 230 aa vs 657 nt and 219 aa, respectively) (Fig. 1).

**Figure 2:**
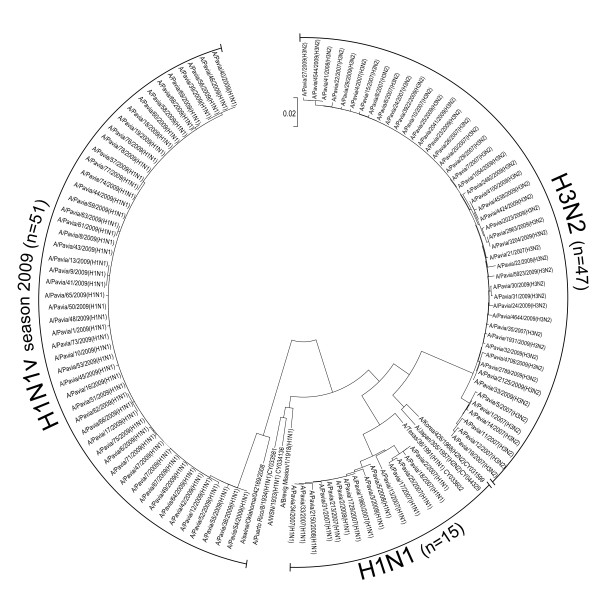
**Phylogenetic tree of NS1 sequences**. Phylogenetic analysis of influenza A NS1 gene in H1N1v isolates with respect to H1N1 and H3N2 isolates from previous seasonal epidemics [Gen Bank:HM745138-HM745250].

The three groups of gene sequences resulted phylogenetically distinct from each other (Fig. 2). Among the H3N2 strains, two NS1 sequence clusters were observed: the first included six isolates from the 2006-2007 season and the second 41 isolates from the 2006-2007, 2007-2008 and 2008-2009 seasons. Similarly, NS1 sequences from seasonal H1N1 strains could be grouped in one cluster including five strains from the 2006-2007 season, one including nine strains from the subsequent season and one sequence from the 2008-2009 season (Fig. 2). No substantial genetic drift was observed in the group of the H1N1v isolates collected during the study period (Fig. 2).

The NS1 sequence from the pandemic H1N1v and seasonal H3N2 and H1N1 strains were then compared with a human and a swine reference sequence (Table 1). The seasonal H1N1 strains showed higher similarity to the human "Spanish" influenza A/Brevig/Mission/1/1918 reference strain (90.9%), than the H1N1v strains (83.4%). In contrast, the pandemic H1N1v strains showed greater similarity to the swine A/Swine/Oklahoma/042169/2008 reference strain (93.8%).

**Table 1:** Nucleotide and amino acid variability of the influenza A NS1 gene H1N1v isolates with respect to H1N1 and H3N2 isolates from previous seasonal epidemics in comparison with reference human (A/Brevig Mission/1/1918) and swine influenza A H1N1 (A/Swine/Oklahoma/042169/2008) isolates.

	Mean % nucleotide identity (range)		Mean % amino acid identity (range)	
**Influenza strains**	**A/Brevig Mission/1/1918**	**A/Swine/Oklahoma/042169/2008**	**A/Brevig_Mission/1/1918**	**A/Swine/Oklahoma/042169/2008**

H1N1	90.9 (90.4-91.5)	81.8 (81.1-82.2)	86.8 (85.4-87.4)	79.2 (78.4-79.5)
H3N2	88.0 (87.5-88.4)	80.0 (79.3-80.4)	82.7 (81.8-83.4)	76.2 (74.2-77.1)
H1N1v	83.4 (83.1-83.7)	93.8 (93.6-94.1)	83.2 (82.2-83.9)	92.1 (91.4-92.9)

### Intra-strain NS1 variability

Intra-strain analysis showed high level of nucleotide and amino acid identity in each group of NS1 sequences (Table 1), and the coefficient of variation (CV) for phylogenetic distances with respect to the mean value of each group of sequences was always <3% (Table 2).

**Table 2:** Intra-strain nucleotide and amino acid variability of influenza A NS1 complete gene and the three functional gene domains of H1N1v isolates with respect to H1N1 and H3N2 isolates from previous seasonal epidemics.

		Mean % nucleotide identity (range)			
**Influenza Strain**	**Max nucleotide substitution**	**Complete Gene**	**Gene Domains**		

			**RNA binding domain (nt 1-219)**	**Effector domain (nt 220-621)**	**Disordered tail (nt 622-657/690)**

H1N1	16	98.9 (97.6-100)	99.0 (97.2-100)	98.8 (97.2-100)	99.1 (97.0-100)

H3N2	13	99.4 (98.1-100)	99.1 (96.3-100)	99.6 (98.2-100)	99.0 (95.5-100)

H1N1v	4	99.8 (99.4-100)	99.8 (99.1-100)	99.7 (99.3-100)	99.9 (99.9-100)

	**Mean % amino acid identity (range)**				

**Influenza Strain**	**Max amino acid substitution**	**Complete Gene**	**Gene Domains**		

			**RNA binding domain (aa 1-73)**	**Effector domain (aa 74-207)**	**Disordered tail (aa 208-219/230)**

H1N1	6	98.6 (96.9-100)	99.6 (97.2-100)	98.2(96.3-100)	97.3(90.9-100)

H3N2	8	98.9 (96.0-100)	98.7 (91.4-100)	99.3 (96.9-100)	97.4 (86.0-100)

H1N1v	4	99.5 (98.6-100)	99.7 (97.2-100)	99.4 (97.7-100)	100

### Inter-strain NS1 variability

Inter-strain analysis showed 87.7% nucleotide identity between H1N1 and H3N2 strains, 78.8% nucleotide identity between H1N1 and H1N1v strains, and 77.9% nucleotide identity between H3N2 and H1N1v strains. Inter-strain amino acid identities between H1N1 and H3N2, H1N1 and H1N1v as well as H3N2 and H1N1v were 84.0%, 78.9% and 77.6%, respectively. Thus, seasonal (H3N2 and H1N1) strains showed a greater nucleotide and amino acid identity between themselves than with respect to the pandemic H1N1v strains (Table 1). However, a statistically significant difference among each of the three groups of sequences was observed (p < 0.001).

The inter-strain analysis performed on the three functional domains of NS1 (RNA-binding domain, codons aa 1-73; effector domain, codons aa 74-207; disordered tail, codons aa 208-219/230) showed a greater nucleotide identity in the effector domain with respect to the RNA binding domain, while the lowest identity was observed in the disordered tail (Table 3). When considering the amino acid identity in the three protein domains, a different picture was observed. In fact, the most conserved domain was the RNA binding domain followed by the effector domain and the disordered tail, respectively (Table 3). Interestingly, all nucleotide and amino acid inter-strain differences (H1N1v vs H3N2, H1N1v vs H1N1 and H1N1 vs H3N2) were statistically significant (p < 0.001).

**Table 3:** Inter-strain nucleotide and amino acid variability of influenza A NS1 complete gene and the three functional gene domains of H1N1v isolates with respect to H1N1 and H3N2 isolates from previous seasonal epidemics.

Mean % nucleotide identity				
**Strains group comparison**	**Complete Gene**	**Gene Domains**		

		**RNA binding domain (nt 1-219)**	**Effector domain (nt 220-621)**	**Disordered tail (nt 622-657/690)**

H1N1 vs H3N2	87.7	84.8	89.5	84.6

H1N1 vs H1N1v	78.8	77.6	80.6	64.7

H3N2 vs H1N1v	77.9	74.9	79.1	80.5

**Mean % amino acid identity**				

**Strains group comparison**	**Complete gene**	**Gene Domains**		

		**RNA binding domain (aa 1-73)**	**Effector domain (aa 74-207)**	**Disordered tail (aa 208-219/230)**

H1N1 vs H3N2	84.0	82.8	86.2	67.2

H1N1 vs H1N1v	78.9	86.1	78.5	41.7

H3N2 vs H1N1v	77.6	81.5	76.5	66.2

### Distribution of NS1 amino acid polymorphisms

In Fig. 1, distribution of NS1 aa polymorphisms in the three groups of virus strains is shown. H3N2 strains showed the wider distribution of aa polymorphisms, with 21 polymorphic codons (21/230, 9.1%) evenly spread over the three functional domains. The H1N1 strains had 12/230 (5.2%) and H1N1v strains had 8/219 (3.7%) polymorphic codons concentrated mostly in the effector domain, even though the effector domain regions carrying polymorphic codons were different in H1N1v vs H1N1 strains (Fig. 1). Among the H1N1v polymorphic codons the V123I substitution was observed in 3/51 (5.8%) strains respect to seasonal H1N1 and H3N2 (15/15 and 47/47, respectively) and N133D substitution was significantly more frequent (13/51, 25.6%) than in seasonal H1N1 and H3N2 strains (0/15 and 0/47, respectively). All gene sequences of H1N1v, H1N1 and H3N2 isolates were identical to those obtained with the corresponding clinical specimens.

## Discussion

NS1 is considered a highly conserved influenza A gene [7]. However, the NS1 evolutionary change rate (as measured by nt substitutions per site per year) is similar to that of the viral HA gene [21].

The 2009 influenza A pandemic will be remembered as the pandemic that did not live up to expectations. For example it was predicted that the pandemic virus would emerge with a new HA or NA subtype and that the emergence of the new virus would be responsible for a severe clinical syndrome and increased mortality due to the lack of pre-existing herd immunity against the new virus subtype. Even though infection by H1N1v virus showed mild clinical symptoms and low mortality, the new virus efficiently spread worldwide despite the presence of strong herd immunity against subtype H1N1, consolidated after decades of seasonal epidemics and vaccination campaigns.

The pandemic H1N1v influenza virus originated from a reassortant Eurasian avian-like swine A/H1N1 virus and a triple-reassortant virus circulating in North American swine [8]. As such, the H1N1v virus contains NA and M genes from Eurasian avian-like swine A/H1N1 virus, and the remaining genes from the triple-reassortant virus - PB2 and PA (avian virus), PB1 (human A/H3N2), and HA, NP and NS (classical swine A/H1N1). The novel H1N1v virus is antigenically distant from the prevailing human H1N1 virus, and there is little prior cross-reacting humoral immunity in the population, with the exception of those individuals older than 60 years [22].

A greater intra-strain variation was observed for seasonal H1N1 and H3N2 variants with respect to H1N1v. However, the latter were collected over a shorter time span.

A statistically significant difference between NS1 sequences of seasonal strains and NS1 of the pandemic strain and each of the two seasonal strains was observed. Thus, the pandemic H1N1v strain was endowed with a highly divergent NS1 gene, with close genetic similarity to the NS1 of the swine reference strain. In addition, the distribution of polymorphic codons clearly differentiated NS1 of H3N2 strains from both H1N1v and H1N1, but important differences were observed also between the H1N1v and seasonal H1N1. Moreover, one marked difference with previous seasonal human influenza strains was the deletion of a carboxy terminal portion of the protein. The observed differences between H1N1v, H1N1 and H3N2 strains could not be attributed to selective pressure on NS1 during culture in MDCK cells since all gene sequences of H1N1v, H1N1 and H3N2 isolates were identical to those obtained with the corresponding clinical specimens.

Although truncated NS1 sequences have been described in human strains [23], the significance of this genetic alteration is still debated. Previous studies have shown that alteration of the C-ter PL domain might be associated with increased virulence in both mammalian [24] and avian [25] strains. A human H1N1 strain with a 7-amino acid extension in the NS1 protein emerged in the '40s circulating until the '80s, when the reverted genotype became prevalent [4]. This extension has been proposed to "mask" the PL-domain, inhibiting its association with cellular PDZ proteins. In addition, Melen et al. [5] have correlated this NS1 alteration with a different nuclear and nucleolar localization of the protein. However, the impact of the 11 amino acid deletion in the NS1 protein of the H1N1v on strain virulence, host adaptation or replicative capacity remains to be further defined.

Our analysis showed that the H1N1v NS1 proteins had a lower number of inter-strain amino acid differences than the NS1 from seasonal H1N1 viruses. Of these differences, six out of eight amino acid changes occurred in the effector domain. In particular, position 123 represents the domain that interacts with the dsRNA-activated protein kinase (PKR), an important modulator of the innate immune response. An artificially induced V123A mutation was shown to reduce this interaction, rendering the virus less efficient in inhibiting the PKR-activated response [26]. None of the other residues has been reported to play a role in protein-protein or protein-RNA interactions, suggesting that they are polymorphisms with no clear functional consequences. Overall, it appears that the H1N1v NS1 protein did not accumulate adaptation-specific mutations during the course of the epidemic, further reinforcing the notion that the original virus was already well-adapted to mammalian hosts. Whether such a stable and well-adapted virus might evolve into a more virulent strain during sustained circulation in the human population remains to be determined.

## Competing interests

The authors declare that they have no competing interests.

## Authors' contributions

GC, SP, FR, EP: sample collection, virus isolation, NS1 gene sequencing. AP, GC: sequence analysis. GC, GM, FB: manuscript preparation. FB: fundraising. All authors read and approved the final manuscript.
